# The Trojan horse - neuroinflammatory impact of T cells in neurodegenerative diseases

**DOI:** 10.1186/s13024-017-0222-8

**Published:** 2017-10-27

**Authors:** Annika Sommer, Beate Winner, Iryna Prots

**Affiliations:** Department of Stem Cell Biology, Friedrich-Alexander University (FAU) Erlangen-Nürnberg, Erlangen, Germany

**Keywords:** Neuroinflammation, Lymphocytes, Parkinson’s disease, Alzheimer’s disease, Multiple sclerosis

## Abstract

Neuronal degeneration is a common mechanism of many neurological diseases including Parkinson’s disease (PD), Alzheimer’s disease (AD), and Multiple Sclerosis (MS). While AD and PD are classical neurodegenerative diseases, the primary pathology in MS is driven by autoimmune inflammation, attacking oligodendrocytes and thereby inducing neurodegeneration. In AD and PD, immune cells are also considered to play an important role in the disease progression. While the role of local central nervous system (CNS) innate immune cells is well described, a potential influence of adaptive immune cells in PD and AD is not yet fully understood.

Here, we aim to summarize findings concerning adaptive immune cells in PD pathogenesis and compare them to AD and MS. In the first part, we focus on disease-specific alterations of lymphocytes in the circulating blood. Subsequently, we describe what is known about CNS-infiltrated lymphocytes and mechanisms of their infiltration. Finally, we summarize published data and try to understand the mechanisms of how lymphocytes contribute to neurodegeneration in PD, AD, and MS.

Lymphocytes are critically involved in the pathogenesis of MS, and clarifying the role of lymphocytes in PD and AD pathogenesis might lead to an identification of a common signature of lymphocytes in neurodegeneration and thus pave the road towards novel treatment options.

## Background

**Fig. 1:**
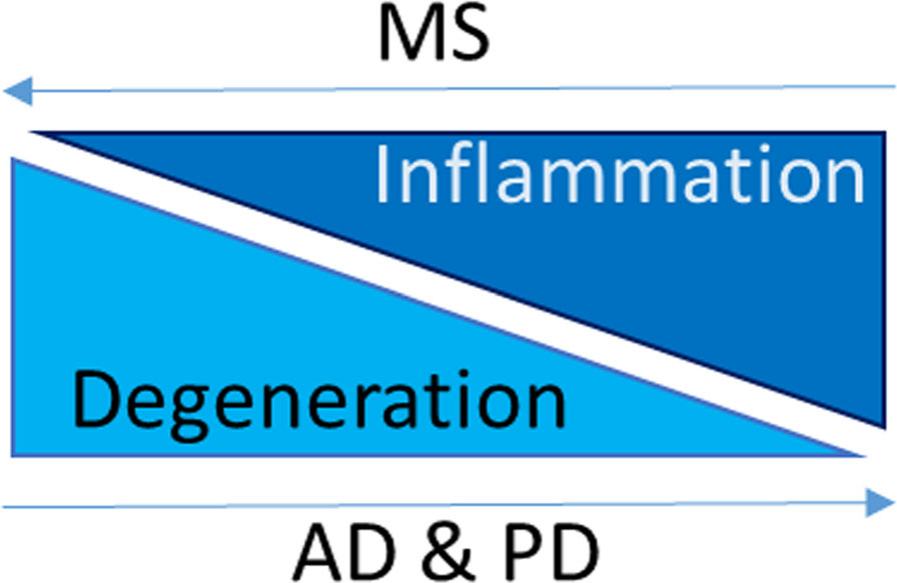
Timeline of classical neurodegenerative diseases and inflammation-driven neurodegenerative disease. In the neurodegenerative diseases AD and PD, neuronal degeneration is the primary pathology, while inflammation is consecutively observed. On the other side, in MS, inflammation is thought to be the primary pathophysiological event, leading to neuronal degeneration. *AD = Alzheimer’s disease; PD = Parkinson’s disease; MS = Multiple Sclerosis*

The two most common neurodegenerative diseases are Parkinson’s disease (PD) and Alzheimer’s disease (AD). PD is clinically depicted by severe motor symptoms including rigidity, postural instability, resting tremor, and bradykinesia [1]. PD pathology is characterized by progressive degeneration and loss of dopaminergic (DA) neurons in the substantia nigra (SN) pars compacta, among other neurons. Moreover, the deposition of α-synuclein as insoluble and toxic aggregates is a characteristic hallmark of PD [2, 3]. AD patients suffer from irreversible loss of memory, progressive cognitive impairment, language disorders, and impairment in their visuospatial skills due to degeneration of hippocampal and cortical neurons, extracellular amyloid plaques and intracellular neurofibrillary tangles [4]. Up to date, the etiology of PD and AD is not fully understood, however inflammation is considered a vital disease process. While the earliest disease pathology in PD and AD is neuronal degeneration, inflammation is consecutively observed, most likely activated by damaged neurons (Fig. 1).

Another common neurologic disease is Multiple Sclerosis (MS), characterized by the progressive loss of neuronal function caused by autoreactive immune cells, resulting in chronic destruction of the axonal myelin sheath in the central nervous system (CNS) [5]. In contrast to PD and AD, in MS, autoimmune inflammation, driven by invading peripheral immune cells, is considered the primary pathophysiological event leading to injury and degeneration of oligodendrocytes and neurons (Fig. 1). We reach out to search for a neuroinflammatory signature of these three diseases with different etiology and pathology course.

While the role of innate immune cells is frequently described in all three here mentioned diseases [6, 7], the contribution of adaptive immune cells is only recognized as essential factor in MS [8]. How and to what extend adaptive immune cells contribute to the pathogenesis of AD and PD remains largely elusive. Here, we review recent data concerning the role of adaptive immunity in PD, focusing on the direct interaction of adaptive immune cells and neurons. The signatures of adaptive immune cells in PD are compared to AD and MS. This knowledge will be strongly relevant for studies exploring blood in search for novel biomarkers for the diagnosis of neurodegenerative diseases or for developing new therapeutic compounds.

## Main text

### Evidence of alterations in circulating T lymphocyte populations

**Fig. 2:**
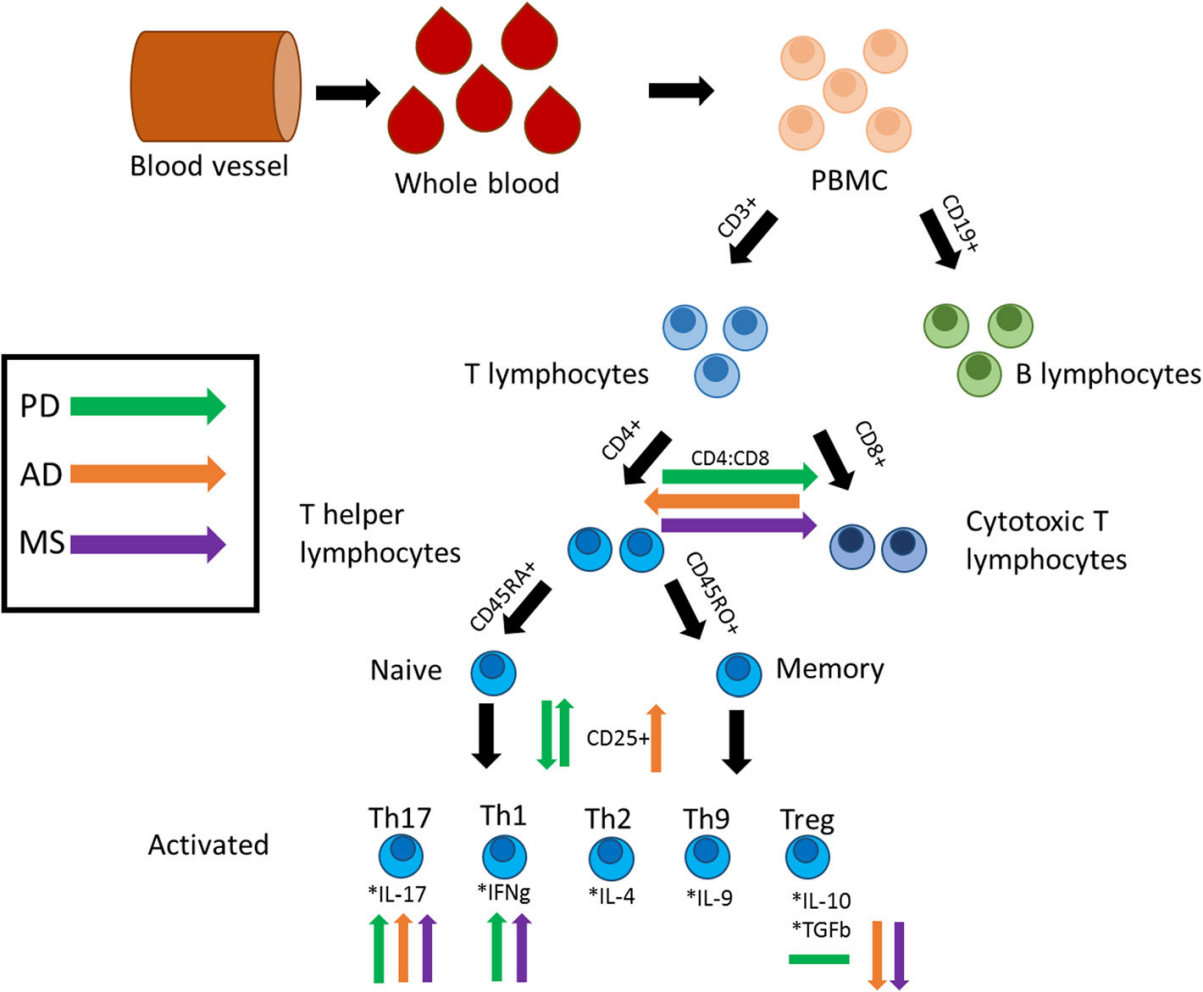
Overview of lymphocytes in the circulating blood and their alterations in neurodegenerative diseases. Peripheral blood mononuclear cells (PBMC) can be isolated from whole blood derived from the blood vessels. PBMC can be further separated into T and B lymphocytes depending on the surface expression of either cluster of differentiation (CD)3 or CD19, respectively. T lymphocyes are further subdivided into T helper cells (CD4+) or cytotoxic T cells (CD8+). Expression of CD4 and CD45RA labels naive Th cells, while expression of CD4 and CD45RO is indicative for memory Th cells. Once activated, Th cells express CD25. Activated Th lymphocytes can be further characterised by the production of cytokines (IL-17, IFNg, IL-4, IL-9, IL-10/TGFb). Disease-specific alterations of the respective lymphocyte subtypes are indicated by arrows (green arrows for PD, orange arrows for AD, and purple arrows for MS). *PBMC = peripheral blood mononuclear cells; CD = cluster of differentiation; Th = T helper cells; IL = Interleukin; IFNg = Interferon gamma; TGFb = Transforming growth factor beta; Treg = regulatory T cells; AD = Alzheimer’s disease; PD = Parkinson’s disease; MS = Multiple Sclerosis*

The cells of the adaptive immune system are the T and B lymphocytes. Activation of lymphocytes and subsequent initiation of an adaptive immune response depends on the presentation of antigens to T lymphocytes. Depending on the respective immune response type, lymphocytes are divided into two classes: 1) B lymphocytes, which initiate an antibody response, and 2) T lymphocytes, which provide a cell-mediated response (Fig. 2). T lymphocytes can be further subdivided into CD8^+^ cytotoxic T (Tc) and CD4^+^ T helper (Th) lymphocytes, depending on the type of their action: either to eliminate infected somatic cells (Tc), or to provide help to and to guide other immune cells (Th) (Fig. 2).

Analysis of lymphocyte populations circulating in the blood of patients or healthy individuals can be performed using whole blood samples, isolated peripheral blood mononuclear cells (PBMC), or isolated B or T lymphocytes (Fig. 2). In 1992, it was described that the isolation of mononuclear cells by density gradient enrichment from whole blood samples can lead to an alteration of lymphocyte subsets [9]. However today, many studies are using PBMC or isolated lymphocytes for ex-vivo analysis [10, 11], indicating that the technical development made it possible to isolate different types of blood cells without interfering with their state.

Analysis of circulating lymphocytes in the blood of PD patients revealed significant alterations in frequencies of lymphocyte subpopulations, indicating an activated adaptive immunity in PD pathogenesis. Several studies demonstrated impaired frequencies of CD4^+^ T cells [12] and reduced CD4^+^: CD8^+^ T cell ratios due to decreased percentages of Th lymphocytes and increased percentages of Tc lymphocytes [13–15], as well as a decrease of CD4^+^ CD45RA^+^ “naive” T cells, and an increase of CD4^+^ CD45RO^+^ “memory” T cells in the blood of 64 [13] and 72 PD patients [12] (Table 1). On note, increased frequencies of memory effector CD45RO^+^ T cells were associated with more severe motor dysfunction in PD patients [12]. Furthermore, Bas and colleagues found an increase in the CD4^+^ CD25^+^ T cell population indicative for systemic immune activation in PD [13]. These findings direct towards an activated, cytotoxic immune signature in PD pathology.

**Table 1:** Summary of T cell populations’ alterations found in PD, AD, and MS patients’ blood

T cell population	PD	AD	MS
CD4: CD8 ratio	Reduced [12–15]	Increased [38]	Reduced [46]
Th1 cells	Reduced [17]	No differences [41]	Increased [47]
Th17 cells	Increased [18]	Increased [41]	Increased [47]
	Comparable [17]		
Treg cells	Comparable [25]	Increased [25]	Reduced [48]
	Impaired function [12]		

Even more indicative for an activated adaptive immune system in PD are findings of alterations in effector Th cell subclasses in the blood of PD patients. The effector Th cell immune response is performed by the release of cytokines. Depending on the cytokines produced, Th cells can be categorized into different effector Th subclasses: pro-inflammatory Th1 (producing IFNg) or Th17 (producing IL-17) cells, anti-inflammatory Th2 cells (producing IL-4) as well as regulatory T cells (Tregs) and the Th9 cells (Fig. 2). In PD, an increased ratio of IFNg- to IL-4-producing Th cells was described in a study investigating 33 PD patients, indicating a shift towards the Th1 type immune response [14]. Of note, IFNg-secreting Th1 cells are responsible for a pro-inflammatory immune response raised by intracellular pathogens and are implicated in a number of autoimmune diseases [16]. The other prominent pro-inflammatory Th effector subpopulation, the Th17 cells, which release the cytokine IL-17, has only been rarely studied in PD until now. One study describes comparable frequencies of Th17 cells (defined as CD4^+^ CD45RO^+^ CCR6^+^) in the peripheral blood of 29 PD patients and 30 healthy individuals [17]. However, defining the Th17 cells by the co-expression of the surface marker CD45RO and CCR6 is not as precise as e.g. measuring the production of IL-17. Quite recently, a study investigated whole blood of 18 early disease PD patients and found elevated frequencies of IL-17-producing CD3^+^ CD8^−^ cells [18]. Emphasizing a potential role of Th17 cells in PD progression, a polymorphism in the IL-17A gene was shown to increase the risk of cognitive impairment in PD [19]. Th17 cells are thought to be strongly involved in autoimmune inflammation [20], thus further studies of Th17 cells in PD patients’ blood would give insight into possible autoimmune inflammatory mechanisms in PD pathology.

The counter players of the pro-inflammatory effector T cell subsets are Treg cells. Treg cells release the anti-inflammatory cytokines IL-10 and TGFb (Fig. 2), downregulate an immune response by suppression of effector T cell function and proliferation and are involved in maintenance of tolerance to self-antigens and prevention of autoimmune diseases [14, 21, 22]. Baba and colleagues defined CD4^+^ CD25^+^ T cells as Tregs and found them to be reduced in 33 PD patients [13, 14]. Notably, a reduction of the Treg cell population is associated with the development of chronic inflammation and autoimmunity [23, 24]. However, to more accurately define the Treg cell population, additional markers such as low expression of IL-7 receptor (CD127) or expression of the Treg-specific transcription factor Forkhead-Box-Protein P3 (FoxP3) should be considered to better interpret the results. Two later studies investigating frequencies of CD4^+^ FoxP3^+^ Treg cells in 30 PD and 33 age-matched controls and of CD4^+^ CD25^+^ CD127^−^ Treg cells in 113 PD and 96 controls did not detect any differences between PD and controls [12, 25]. Interestingly, although not altered in quantity, Tregs of PD patients were significantly impaired in their ability to suppress proliferation of effector T cells [12].

Partly inconsistent results in the studies described above may arise due to the heterogeneity of PD patients involved as well as different selection criteria for patients and control individuals. PD is a progressive disease, thus the recruited patients could suffer from different disease stages, potentially affecting the results. Furthermore, the origin of PD may vary between patients: while most patients suffer from a sporadic disease, there are about 18 genes known to cause familial forms of PD. Though, genetic mutations influence the disease progression [26] and might modulate the lymphocyte populations as well [27, 28]. In addition, it was proposed that medication could influence the lymphocyte populations in the peripheral blood [29]. A frequent treatment option for PD patients is levodopa, which could also affect T lymphocytes by binding to dopamine receptors (DR) expressed on T lymphocyte cell surface [30]. Indeed, levodopa was associated with a reduced number of peripheral IFNg-producing Th cells [31]. However, another study was able to correlate T lymphocyte alterations with the clinical stage of the disease (Hoehn & Yahr stage) rather than with levodopa medication [15]. Thus, different factors, including disease progression, age, medication, or alternative isolation technics might influence the outcome of the blood studies in PD patients. In future experiments, the involvement of these factors should be investigated in more detail and studies should be performed under comparable standards to allow the generation of valid results.

Besides alterations in T lymphocytes, a reduction of B lymphocytes [13–15] as well as an increase in natural killer (NK) cells [17] was described in PD patients. Additionally, several autoantibodies directed against antigens associated with PD pathogenesis have been identified, including antibodies against melanin [32], α-synuclein [33], and GM1 ganglioside [34, 35]. Importantly, a recent study by Sulzer and colleagues could show that α-synuclein peptides and protofibrils act as antigenic epitopes for T lymphocytes from PD patients and might thereby drive adaptive immune response [36]. Specifically, this study has reported activation of IL-5-producing and IFNg-producing effector T cell subsets in response to α-synuclein in PD patients, suggesting that autoimmune inflammation driven by imbalanced effector T cell populations might take place in PD pathogenesis. However, further analyses are required to accurately define and validate profiles of cytokine-producing T cells after exposure to α-synuclein as potential autoreactive T cell subsets in pathogenesis of PD [37].

Interestingly, investigations of lymphocyte populations in the blood of AD patients partially led to results comparable to findings in PD patients (Table 1). The frequency of CD4^+^ is increased and the frequency of CD8^+^ T cells is decreased in AD patients [38, 39]. This is in contrast to the findings in PD. A reduced CD45RA^+^ and increased CD45RO^+^ Th cell populations were described in 40 AD patients, resembling the findings in PD [40]. Additionally, an increase in CD4^+^ CD25^+^ activated T cells (FoxP3^−^) [40] was found in 40 AD patients, which is also described in PD patients [13]. Studies investigating the Th effector populations in 38 AD patients revealed no differences in IFNg-producing CD4^+^ T cells [41]. However, an increase in Th17 cells was present, also documented by elevated frequencies of the transcription factor RAR-related orphan receptor gamma t (RORyt) and enhanced production of IL-22 (a cytokine produced partly by Th17 cells) by CD4^+^ T cells in flow cytometry analysis [41]. Treg cells, characterized by the expression of CD4^+^ and FoxP3^+^, were described to be increased in 23 AD patients [25]. Besides, increased IL-9-producing Th9 cells were found in AD patients’ blood. IL-9 is involved in tissue inflammation and can either be inflammatory or regulatory depending on the context and the source of the producing cells [42].

Thus, blood studies in AD patients indicate modulations of adaptive immune cells. While, on one side, increased numbers of Th17 cells indicate a pro-inflammatory signature, on the other side, increased frequencies of Treg cells indicate an upregulation of anti-inflammatory mechanisms in AD. Future studies should clarify the role of adaptive immune cells in AD.

Furthermore, in accordance to the findings in PD patients’ blood, increased numbers of circulating autoantibodies against amyloid beta were found in the blood of AD patients [43–45].

In the inflammation-driven neurodegenerative disease MS, T lymphocyte populations have been a longstanding matter of research. Reduction of both, total CD3^+^ and CD4^+^ T cells were found in the peripheral blood of MS patients [46]. Analysis of the Th cell effector populations revealed increased levels of IFNg and IL-17, key cytokines of the pro-inflammatory Th1 and Th17 cell populations, respectively, [47]. In addition, impaired Treg cell function accompanied by decreased FoxP3 expression [48] in the blood of MS patients was found. A reduction of Treg cells combined with an increased Th1/Th17 cell population is thought to be a cause of the imbalanced immune tolerance in MS, leading to increased disease susceptibility and influencing the course of autoimmunity.

In accordance with MS pathology, in AD and PD, increased frequencies of Th17 cells were described, indicating a common pro-inflammatory signature in all here described neurodegenerative diseases. This suggests a connection between activation of adaptive immune cells and neurodegeneration, with an important role of Th17 cells. However, while the Th17 cell-mediated pro-inflammatory immune response is well established in MS, only limited studies, showing partly controversial results, described Th17 cells in AD or PD. Thus, future studies should concentrate on the role of Th17 cells in AD, PD, and other neurodegenerative diseases, in order to better understand the contribution of this subtype of adaptive effector cells in the pathogenesis of these diseases.

Further analysis should also clarify, if alterations of effector T cell populations found in the blood of the patients are also present in the CNS within characteristic neurodegenerative regions or in the close proximity of affected neuronal populations. To our knowledge, no data on profiling of effector T cell subsets in the CNS of PD patients have been published yet. Alternatively, detection of effector T cell subset-specific cytokines could give a hint for the composition of T cell subsets in the CNS of PD patients. Whereas elevation of innate pro-inflammatory cytokines (IL-1b, IL-6, TNFa, TGFa, TGFb1, EGF) was reported in the brain or cerebrospinal fluid (CSF) of PD patients [49, 50], only an increase of two T cell-associated cytokines IL-4 in PD brain tissues [50] and IFNg in the CSF of cognitively impaired PD [51] was demonstrated. However, limited amount of data does not allow making conclusion about effector T cell subsets within the CNS of PD patients. Further studies will be necessary to define cytokine profiles of IFNg, IL-4, IL-17, IL-9 and the prevalence of effector Th subsets in the CNS of PD patients, allowing the comparison with the peripheral blood findings.

Besides being used as a tool to identify potential contribution of systemic inflammation in neurodegenerative cascades, peripheral immune cell constellations could also function as biomarker, allowing diagnosis before onset of the clinical symptoms. Therefore, proteome alterations in T lymphocytes [52, 53] or plasma [54] were used to build predictive models aiming to identify potential biomarkers in neurodegenerative diseases early in the disease course. In PD, Alberio and colleagues identified two proteins differentially expressed in T lymphocytes (ß-fibrinogen and transaldolase, [52]) and nine different proteins with differential expression in the plasma [52, 54] of PD patients as potential biomarkers for the disease duration and severity. In AD, differences in peripheral blood lymphocyte proliferation activity were used to develop a model that might be utilized as an additional AD diagnostic tool [53].

### Infiltration of T lymphocytes into the CNS of PD, AD, and MS patients

The alterations of T lymphocyte populations in the peripheral blood of PD patients are indicative of a contribution of T lymphocytes to PD pathology. To study this in more detail, infiltration of T lymphocytes into the CNS was investigated in animal PD models and human postmortem brain tissues. Indeed, in 1988, a study illustrated CD3^+^ staining (a general T lymphocyte marker, Fig. 2) within the CNS of parkinsonian brains, pinpointing T lymphocyte CNS-infiltration and the contribution to disease progression [55]. In accordance, Brochard and colleagues showed staining of CD4^+^ and CD8^+^ cells, indicative for Th and Tc cells, respectively, in the SN of postmortem tissue from PD patients, while B and NK cells were not found [48]. The CNS-infiltrating CD4^+^ and CD8^+^ cells were shown to be in close contact to blood vessels or in the vicinity of melanized DA neurons in the SN but not present in the red nucleus, which is not affected in PD [56]. This suggests that infiltration of T lymphocytes might be site-specific rather than systemic, and connected to areas of PD pathology. Subsequently, in the toxin-induced 1-methyl-4-phenyl-1,2,3,6-tetrahydropyridine (MPTP) mouse model of PD, CD4^+^ and CD8^+^ cells were found in the midbrain and were associated with nigrostriatal neuronal cell death [56]. Furthermore, we demonstrated positive staining for CD3^+^ in the midbrain of the transgenic synucleinopathy model of PD (mice overexpressing human wild-type α-synuclein [WTS] under the Thy1 promoter [Thy1-WTS]) [57]. By now, a number of other studies, utilizing different animal models of PD, illustrated positive staining of T lymphocyte-associated markers in PD-relevant CNS regions [58]. While the numbers of CD4^+^ and CD8^+^ T lymphocytes found in postmortem tissue of PD patients are quite low (0.3 CD4^+^ cells/mm^2^ and 1.2 CD8^+^ cells/mm^2^ [56]), up to 142 CD3^+^ T cells were found in the hippocampus of AD patients with an increased number of CD8^+^ cells compared to CD4^+^ cells [59]. An even more prominent T cell infiltration is found in the inflammation-driven neurodegenerative disease MS, in which up to 160 CD3^+^ T cells were detected in active lesions of MS patients [60]. Remarkably, the highest number of infiltrating CD3^+^ T lymphocytes in MS consists of the CD8^+^ Tc cell subtype, while the CD4^+^ Th subtype is in minority [61].

Although many studies demonstrated the presence of T lymphocyte-associated markers in neurodegenerative diseases, the mechanisms of T lymphocyte infiltration remain elusive. Different potential locations, where lymphocytes could invade the CNS, include the blood brain barrier (BBB) and the blood CSF barrier (BCSFB). The BCSFB is formed by the choroid plexus (CP). The CP consists of an epithelial monolayer, which is vascularized by blood vessels and functions as CSF producer, nutrient provider for the CNS and mechanical protection [62]. In contrast to the BBB, which is composed of endothelial cells and astrocytes, the BCSFB is restricted to epithelial cells. Its tight junctions are relative permissive for immune cells to cross. Furthermore, the CP expresses adhesion molecules and chemokines, which support lymphocyte trafficking [63], suggesting that the CP functions as a suitable gateway for lymphocyte entry. In accordance, ultrastructural changes within the CP and upregulation of adhesion molecules (VCAM, ICAM) were found in the experimental autoimmune encephalomyelitis (EAE) mouse model of MS [64] as well as in elderly and AD patients [65]. Not much is known about the role of the CP as gateway for lymphocyte entry in PD.

On the other side, the BBB could serve as a route for lymphocytes to enter the CNS. Since the BBB is formed by endothelial cell tight junctions and astrocytes, which are rather intolerant for lymphocyte trafficking, a dysfunctional BBB would be required to allow lymphocytes to invade the CNS. In PD patients, an increased permeability of the BBB was shown by infiltration of [11C]-verapamil into the CNS of PD patients using positron emission tomography [47]. In accordance, in AD, BBB disruption is a widely accepted pathological feature, suggested to be induced by amyloid beta in the vasculature [48]. In animal models of AD, the expression of the tight junction proteins, zonula occludens (ZO-1) and occluding (important players in the formation of the BBB), was downregulated after ß-amyloid deposition, probably contributing to the BBB dysfunction [49, 50]. In an AD in vitro model, decreased expression of the same tight junction proteins was associated with efficient transmigration of Th17 cells across BBB endothelial cells [51]. In MS, disruption of the BBB by injury of blood vessels is an early event in the disease pathology facilitating massive infiltration of lymphocytes into the CNS [66]. Damage of the BBB in MS has been demonstrated by magnetic resonance imaging as well as by immunohistochemical studies showing tight junction changes and increased influx of plasma proteins [52–55].

Beside lymphocyte invasion into the CNS, the cytokines, released by lymphocytes, could also migrate through the BBB. It was described that in neuronal diseases, pro-inflammatory cytokines are able to cross the BBB and thus to invade the CNS [67].

### Potential effects of T lymphocytes in the pathophysiology of PD, AD and MS

Lymphocyte populations are altered in the periphery and invade the CNS in PD, AD and MS. In the last part of this review, we focus on findings concerning the mechanisms, by which CNS-infiltrating lymphocytes contribute to the disease progression and to neuronal degeneration.

The biggest challenge, investigating the mechanisms of how lymphocytes could contribute to neurodegeneration in the CNS, comprises the limitation of model systems. In humans, only postmortem tissues can be used, which describe the end-stage of the disease and do not give insights into the pathogenesis during disease progression. In future, appropriate human model systems for neurodegeneration should be established, allowing analysis of the inflammatory mechanisms during ongoing neurodegeneration in PD, AD and MS. As alternative approach, animal models are frequently used to investigate the role of CNS-infiltrating T lymphocytes. Although animal models only partly resemble the human disease pathology, they allow analysis of early disease time points in a living system.

**Fig. 3:**
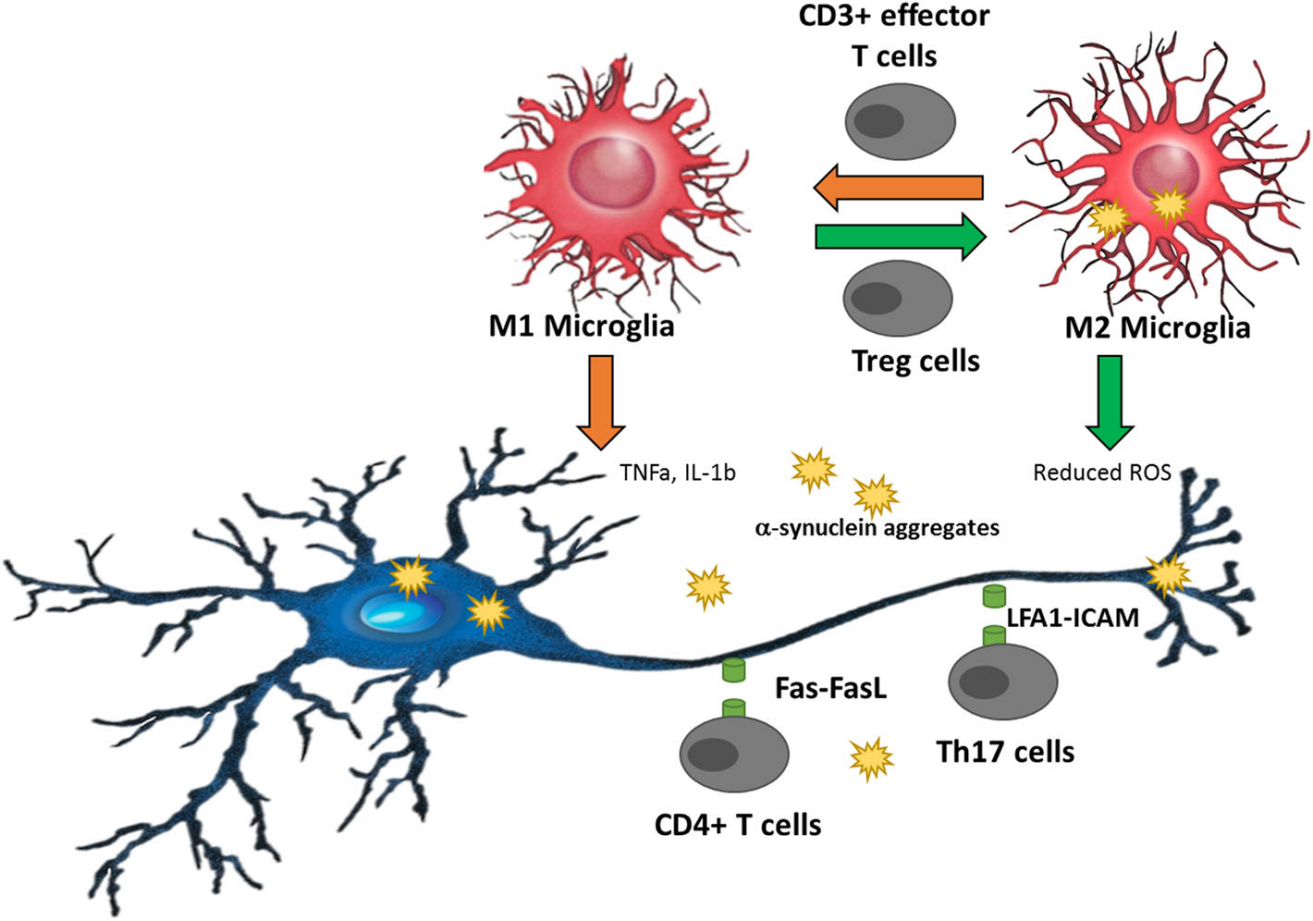
Potential mechanisms, by which T cells contribute to PD pathogenesis. Different mechanisms were recently described, by which T cells could contribute to PD pathogenesis, including direct and indirect interaction of T cells with neurons. Direct interactions could be mediated by Fas-FasL signaling [56] or by interaction of LFA1 with ICAM [68]. Besides, T cells could indirectly contribute to PD pathogenesis by influencing the microglial phenotype, shifting an anti-inflammatory (M2 Microglia) phenotype to a pro-inflammatory (M1 Microglia) or vice versa [57]. Moreover, α-synuclein aggregates might be presented to and activate T lymphocytes [36], thereby triggering an autoimmune inflammation, which in turn would exacerbate PD pathology possibly by imbalance of effector Th subsets, such as Treg vs. Th17 cells [69, 70]

Until now, several publications described different effects of CNS-infiltrating T lymphocytes on PD pathogenesis by using different PD animal models as shown in Fig. 3. Brochard and colleagues demonstrated that Th cells rather than Tc cells induce neuronal death in the SN via Fas/Fas Ligand signaling in the MPTP model of PD [56]. In the Thy1-WTS transgenic mouse model, characterized by pronounced synucleinopathy, we previously showed that CNS-invading CD3^+^ T lymphocytes contribute to the PD pathogenesis by modulation of CNS myeloid cell activation [57]. Within the CNS, T lymphocytes mediated the switch from the anti-inflammatory M2 microglial phenotype to the pro-inflammatory M1 phenotype, thereby reducing their phagocytic activity and clearance of α-synuclein aggregates and thus enhancing α-synuclein pathology [57]. Interestingly, a recent study suggested a role of Th17 cells in neuronal degeneration in the MPTP model [68]. The authors claimed that Th17 cells induce neurotoxicity by directly contacting neurons via adhesion molecules, LFA-1 and ICAM-1, expressed on Th17 cells and neurons, respectively [68].

Yet, T lymphocytes could also mediate a neuroprotective immune response in neurodegenerative disease. In the MPTP model of PD, adoptively transferred activated Treg cells led to an increased protection against MPTP-induced neurotoxicity [69]. Accordingly, in vitro, Treg cells suppressed microglial release of reactive oxygen species (ROS) and thus prevented ROS-induced neuronal damage [70]. In humans, a recent phase 1 clinical trial investigated the effects of a drug sargramostim in a small cohort of PD patients. Sargramostim (human recombinant granulocyte-macrophage colony stimulating factor) is approved as an immunomodulatory drug for patients receiving bone marrow transplantation and was shown to induce Treg immune responses [71]. In PD patients, sargramostim treatment led to increased numbers and improved function of Treg cells along with modest improvement of the unified PD rating scale (UPDRS) III and improved magnetoencephalography-recorded cortical motor activities [72]. This data support an important role of T lymphocytes in PD pathogenesis and potentiate the hypothesis that imbalance between pro- and anti-inflammatory effector T cell subsets contributes to PD.

Besides in vivo and in vitro experiments, human genetics and systems biology approaches provide further evidence of lymphocyte involvement in PD pathogenesis. In recent years, genome-wide association studies (GWAS) identified a number of independent genetic variants and single nucleotide polymorphisms (SNPs) conferring a risk for PD [73–75]. Among 26 loci with significant risk for PD found by GWAS, a strong association of variants in the Human Leukocyte Antigen (HLA) region indicates their direct effect on PD etiology [69, 70]. This genetic region contains multiple major histocompatibility (MHC) class II and class I genes, which play an important role in the antigen presentation to T lymphocytes. Importantly, the biological significance of the association of HLA variants with PD pathogenesis was partly clarified by the study of Sulzer and colleagues [36], showing that MHC molecules associated with PD risk are able to display α-synuclein peptides. Moreover, genes carrying PD risk variants were shown to be enriched for gene sets active in adaptive immune system, in particular in activated Th cells and peripheral Treg cells [75].

In AD, differential effects of CNS-infiltrating T cells were described. On one side, a neuroprotective effect was suggested, by showing that T cells release neurotrophic factors, activate the phagocytosis activity of microglial cells and thus help to reduce amyloid beta deposition (as reviewed in [65]). On the other side, amyloid beta-reactive T cells were shown to boost the AD progression by the release of pro-inflammatory cytokines, causing chronic inflammation [65]. In accordance, IFNg, released by Th1 cells, was shown to increase microglial activation, amyloid beta deposition and led to impaired cognitive function in the amyloid precursor protein/presenilin 1 (APP/PS1) AD mouse model [76]. Treatment of the APP/PS1 mice with an anti-IFNg antibody reduced the disease severity, suggesting that IFNg has a neurotoxic effect in AD pathology probably mediated by activated microglia [76]. Until now, it remains elusive if T cells cause either beneficial or delirious effects in AD progression. Indeed, it is proposed that the T cell profile might depend on the AD stage, with increased pro-inflammatory reactions as the disease progresses [65].

Besides, systems biology approaches highlighted an adaptive and innate immune signatures and microglia-specific module in transcriptional and genotyping profiles of AD postmortem tissues [75, 77].

Detailed studies of how lymphocytes contribute to neuronal injury were performed in MS. In the EAE animal model of MS, infiltrating T lymphocytes were shown to interact with neurons and thereby cause neuronal injury and subsequent neurodegeneration [78]. Tanabe and colleagues recently demonstrated that Th17 cells led to neuronal death in the EAE model due to an interaction of the repulsive guidance molecule-a (RGMa) on Th17 cells with neogenin on neurons followed by dephosphorilation of Akt [79]. Blockage of RGMa reduced EAE severity and neuronal degeneration without affecting immune or glial responses [79]. In accordance, a fine mapping approach of GWAS-identified loci with MS risk could clearly map disease-associated variants with high causative probability to immune enhancers [80].

Interestingly, Gagliano and colleagues compared genomic data from MS patients to AD and PD patients and detected significant heritability enrichments for annotations marking innate and adaptive immune cells in MS and to a lower degree in AD. In PD, the strongest signals were found in annotations marking adaptive immune system [75]. Further systems biology analysis and comparison of genomics of different T cell-associated diseases could help to clarify T cell-mediated effects in neurodegenerative diseases like PD or AD.

## Conclusion

Taken together, an important contribution of adaptive immune cells to the pathophysiology of neurodegeneration in PD is evident with the majority of studies showing involvement of T lymphocytes. The evidence of an activated adaptive immune system is not only found in typical inflammation-driven neurodegenerative disorder MS, but also in classical neurodegenerative disorders like PD and AD [75]. Neurodegeneration is linked to neuroinflammation via an imbalance of pro-inflammatory effector T cells (releasing e.g. IFNg or IL-17) and Tregs, leading to neuronal damage and reduced neuroprotective effect. Thus, developing compounds that promote expansion of anti-inflammatory, neuroprotective Treg cells might be a promising approach, aiming to reduce the progression of neurodegenerative diseases like PD. Furthermore, developing compounds capable of targeting peripheral immune cells rather than CNS innate immune cells might simplify the drug development since drugs would not need to cross the BBB.

However, most of here described studies were performed in postmortem tissues or animal models. To study the effect of adaptive immune cells on neurons during the disease progression, suitable human models need to be established. Recent advances in human induced pluripotent stem cell (hiPSC) technology provide a promising approach to bridge the gap between mouse models and human disease. HiPSC-based models generated from PD patient’s cells were already published, recapitulating features of PD pathology, including neurite degeneration [81–83]. Thus, utilizing hiPSC-based PD models to investigate the role of immune cells will provide a suitable tool to deepen the knowledge about immune cells – neurons interactions in human pathology [27].

## Abbreviations

AD: Alzheimer’s Disease
APP/PS1: Amyloid Precursor Protein/ Presenilin 1
BBB: Blood Brain Barrier
BCSFB: Blood Cerebrospinal Fluid Barrier
CD: Cluster of Differentiation
CNS: Central Nervous System
CP: Choroid Plexus
CSF: Cerebrospinal Fluid
DA: Dopaminergic
DR: Dopamine Receptor
EAE: Experimental Autoimmune Encephalomyelitis
FDA: Food and Drug Administration
FoxP3: Forkhead-Box-Protein P3
GM-CSF: Granulocyte-macrophage colony stimulating factor
GWAS: Genome-wide association study
hiPSC: Human induced Pluripotent Stem Cells
HLA: Human leukocyte antigen
IFNg: Interferon g
IL: Interleukin
MHC: Major histocompatibility complex
MPTP: 1-methyl-4-phenyl-1,2,3,6-tetrahydropyridine
MS: Multiple Sclerosis
NK: Natural Killer
PBMC: Peripheral Blood Mononuclear Cells
PD: Parkinson’s disease
RGMa: Repulsive Guidance Molecule-a
RORyt: RAR-related orphan receptor gamma t
ROS: Reactive oxigen species
SN: Substantia Nigra
SNP: Single nucleotide polymorphism
Tc: Cytotoxic T cell
TGFb: Transforming Growth Factor ß
Th: T helper cell
Treg: Regulatory T cell
UPDSR: Unified Parkinson’s Disease Rating Scale
WTS: Wild-type α-synuclein
ZO-1: Zonula Occludens
